# Management of asymptomatic severe aortic stenosis: a systematic review and meta-analysis

**DOI:** 10.1136/openhrt-2022-001982

**Published:** 2022-05-12

**Authors:** Vasiliki Tsampasian, Ciaran Grafton-Clarke, Abraham Edgar Gracia Ramos, George Asimakopoulos, Pankaj Garg, Sanjay Prasad, Liam Ring, Gerry P McCann, James Rudd, Marc R Dweck, Vassilios S Vassiliou

**Affiliations:** 1 Cardiology, Norfolk and Norwich University Hospital, Norwich, UK; 2 Norwich Medical School, University of East Anglia, Norwich, UK; 3 Departamento de Medicina Interna, Centro Medico Nacional "La Raza", IMSS, Ciudad de Mexico, Mexico; 4 Seccion de Estudios de Posgrado e Investigacion, Escuela Superior de Medicina, Instituto Politecnico Nacional, Mexico City, Mexico; 5 Cardiology, Royal Brompton and Harefield NHS Trust, London, UK; 6 School of Medicine, Imperial College London, London, UK; 7 Cardiology, West Suffolk Hospital NHS Trust, Bury Saint Edmunds, UK; 8 Department of Cardiovascular Sciences, University of Leicester, Leicester, UK; 9 Leicester NIHR Biomedical Research Centre, Glenfield Hospital, Leicester, UK; 10 Division of Cardiovascular Medicine, University of Cambridge, Cambridge, UK; 11 British Heart Foundation Centre for Cardiovascular Science, University of Edinburgh, Edinburgh, UK

**Keywords:** meta-analysis, aortic diseases, aortic valve stenosis

## Abstract

**Objectives:**

The management of severe aortic stenosis mandates consideration of aortic valve intervention for symptomatic patients. However, for asymptomatic patients with severe aortic stenosis, recent randomised trials supported earlier intervention. We conducted a systematic review and meta-analysis to evaluate all the available data comparing the two management strategies.

**Methods:**

PubMed, Cochrane and Web of Science databases were systematically searched from inception until 10 January 2022. The search key terms were ‘asymptomatic’, ‘severe aortic stenosis’ and ‘intervention’.

**Results:**

Meta-analysis of two published randomised trials, AVATAR and RECOVERY, included 302 patients and showed that early intervention resulted in 55% reduction in all-cause mortality (HR=0.45, 95% CI 0.24 to 0.86; I^2^ 0%) and 79% reduction in risk of hospitalisation for heart failure (HR=0.21, 95% CI 0.05 to 0.96; I^2^ 15%). There was no difference in risk of cardiovascular death between the two groups (HR=0.36, 95% CI 0.03 to 3.78; I^2^ 78%). Additionally, meta-analysis of eight observational studies showed improved mortality in patients treated with early intervention (HR=0.38, 95% CI 0.26 to 0.56; I^2^ 77%).

**Conclusion:**

This meta-analysis provides evidence that, in patients with severe asymptomatic aortic stenosis, early intervention reduces all-cause mortality and improves outcomes compared with conservative management. While this is very encouraging, further randomised controlled studies are needed to draw firm conclusions and identify the optimal timing of intervention.

**PROSPERO registration number:**

CRD42022301037.

**Key questions**

**What is already known about this subject?**

- According to the current guidelines and recommendations, patients with asymptomatic severe aortic stenosis are approached with the watchful waiting strategy unless signs of adverse prognosis or rapid disease progression are present.
- The optimal timing of intervention has been debated as evidence suggests that when the adverse signs occur these may be irreversible and therefore it is already too late.
- Recently, the largest randomised controlled trial was published comparing early intervention versus conservative management in this patient group.

**What does this study add?**

- This systematic review and meta-analysis was conducted to critically assess the strengths and disadvantages of the two published randomised controlled trials on the subject.
- Additionally, all observational studies in the literature were evaluated.
- Within the limitations of the trials and the studies, early intervention appears to be associated with a favourable prognosis and lower all-cause mortality rates.

**How might this impact on clinical practice?**

- The irreversible manifestations of severe aortic stenosis may occur before the presentation of symptoms or detectable myocardial impairment, which in turn may preclude worse prognosis and adverse outcomes.
- The results of this meta-analysis may herald the beginning of change in the management of these patients.
- Nevertheless, ongoing trials investigating this high-risk population are anticipated to shed more light into the matter and the identification of the optimal time of intervention.

## Introduction

Aortic stenosis is the valvular heart lesion with the highest clinical impact and mortality worldwide, accounting for half of valve-related deaths.^1 2^ For patients with symptomatic severe aortic stenosis, European and American guidelines indicate that aortic valve replacement is advised as a class IB and class IA recommendation, respectively.^3 4^ However, more ambiguity exists in the management of patients with asymptomatic severe aortic stenosis, where intervention is not recommended unless signs of adverse prognosis are present. These indicators include rapid progression of the valve stenosis, severe valve calcification, valve parameters reflecting the ‘very severe’ end of the spectrum of the disease, or signs of left ventricular impairment by echocardiographic data or blood biomarkers, although strong evidence for these is lacking.^3 4^

However, many would argue that by the time the left ventricular myocardium shows direct or indirect signs of impairment, it is already too late, with irreversible damage established^5^ and the ‘therapeutic window’ for intervention missed. Additionally, studies have shown that most patients with severe asymptomatic aortic stenosis exhibit symptoms in the first 2 years following the diagnosis. Many deaths however occur in the period of ‘watchful waiting’.^6 7^ These observations have caused much controversy among clinicians and highlight the need for improved risk stratification models and better individualised management strategies according to each patient’s risk.

The optimal timing of aortic valve intervention in asymptomatic patients with severe aortic stenosis is yet to be ascertained. Undoubtedly, after the results of the Aortic Valve ReplAcemenT versus Conservative Treatment in Asymptomatic SeveRe Aortic Stenosis (AVATAR) trial,^8^ many would argue whether the time for a change in clinical practice is fast-approaching. We therefore conducted a systematic review and meta-analysis of randomised and observational studies comparing early intervention versus conservative management of patients with asymptomatic severe aortic stenosis to synthesise and evaluate the current evidence.

## Methods

This systematic review and meta-analysis was conducted and reported according to the Preferred Reporting Items for Systematic Reviews and Meta-Analyses guidelines^9^ and has been submitted and registered with PROSPERO (registration number: CRD42022301037). We performed a focused review and meta-analysis of all the randomised trials in the literature comparing aortic valve intervention with conservative management in patients with asymptomatic severe aortic stenosis. The primary endpoints of this meta-analysis included all-cause mortality, cardiovascular death and hospitalisation for heart failure. Additionally, using the same search results we separated the observational studies and performed a meta-analysis of all the published observational studies comparing outcomes, identified through the same search, between early intervention and conservative management in patients with asymptomatic severe aortic stenosis, with all-cause mortality being the primary endpoint.

### Search strategy

PubMed, Cochrane and Web of Science databases were systematically searched from inception until 10 January 2022. The terms used for the search were ‘asymptomatic’, ‘severe aortic stenosis’ and ‘intervention’. After removing duplicates, two investigators independently screened the remaining studies at the title/abstract level (VT, CG-C). Randomised controlled studies comparing mortality and clinical outcomes in patients with asymptomatic severe aortic stenosis treated with early intervention versus conservative management were included in the meta-analysis. Observational studies that compared mortality between early intervention and conservative management in patients with asymptomatic severe aortic stenosis were evaluated separately. The selected studies underwent full-text screening, performed by two independent investigators (VT, CG-C). The following inclusion and exclusion criteria were applied:

#### Inclusion criteria

- Studies published in English and limited to humans.
- Studies with participants >18 years old.
- Randomised controlled studies and observational studies comparing mortality and clinical outcomes in patients with asymptomatic severe aortic stenosis treated with early intervention versus conservative management.

#### Exclusion criteria

- Studies published in language other than English.
- Studies including patients with symptomatic aortic stenosis.

Conflicts were resolved by discussion with a third investigator (VSV), after which consensus was achieved. The primary outcomes of interest were all-cause mortality, cardiovascular death and hospitalisation for heart failure. The study selection process is shown in online supplemental figure 1.

10.1136/openhrt-2022-001982.supp1 Supplementary data

### Data analysis

The HR and 95% CI reported in each study were used for the meta-analysis. A random-effects model with inverse-variance weights was used to combine the effect measures from all studies on a logarithmic scale. Statistical heterogeneity was assessed using I^2^ statistics. Statistical analyses were conducted using the Review Manager (RevMan) software (V.5.3; Copenhagen: The Nordic Cochrane Centre, The Cochrane Collaboration, 2014). Where appropriate funnel plots are also given. Statistical significance was defined as p<0.05.

### Patient and public involvement

Given that this is a systematic review and meta-analysis of already published data, patients or the public were not involved in the design and conduct of this study.

## Results

The search yielded 1046 studies. After removing the duplicates and applying the inclusion and exclusion criteria, 71 articles underwent full-text evaluation. Out of these, two randomised controlled trials, the AVATAR trial^8^ and the Randomized Comparison of Early Surgeryversus Conventional Treatment in Very SevereAortic Stenosis (RECOVERY) trial,^10^ with a total of 302 patients, met the inclusion criteria and their data were extracted for meta-analysis. Of these patients, 151 were randomised to the early intervention group and 151 were randomised to the conservative management group. The two trials included patients with asymptomatic severe aortic stenosis of different aetiologies. The majority of the patients (85%) in the AVATAR trial had degenerative aortic valve stenosis. In contrast, only 33% of the patients had degenerative aortic stenosis in the RECOVERY trial and 61% had bicuspid aortic valve disease. Additionally, the inclusion criteria of the two trials were slightly different, as in the RECOVERY trial patients with very severe aortic stenosis (as evidenced by aortic valve area of <0.75 cm^2^, maximal velocity across the aortic valve (Vmax) >4.5 m/s or mean gradient >50 mm Hg) were included and an exercise test was performed only in selected cases. The main features of the two studies are summarised in table 1, and the baseline demographic and echocardiographic patient characteristics of the two trials are shown in table 2. The risk of bias assessment of these two trials is demonstrated in online supplemental table 1.

**Table 1:** Main characteristics of the two randomised controlled trials

	AVATAR	RECOVERY
Trial design	Multinational, randomised, controlled, parallel-group, event-driven	Multicentre, randomised, controlled, parallel-group, open-label
Recruitment sites	Nine medical centres, seven European Union countries	Four medical centres, one country
Recruitment period	June 2015–September 2020	July 2010–April 2015
Follow-up period (median)	32 months	73 months
Inclusion criteria	Asymptomatic patients. Severe AS (AVA <1 cm^2^, Vmax >4 m/s or MG >40 mm Hg). Negative exercise tolerance test.	Asymptomatic patients. Very severe AS (AVA <0.75 cm^2^, Vmax >4.5 m/s or MG >50 mm Hg). *Exercise testing was selectively performed to evaluate patients with non-specific symptoms (24 patients) and those with a positive exercise test were excluded (3 patients).*
Main exclusion criteria	Symptoms (exertional dyspnoea, syncope, presyncope or angina). LVEF <50%. Very severe AS (>5.5 m/s at rest). Clinically significant aortic regurgitation or mitral valve disease. Significant aortic root and/or ascending aorta dilatation requiring surgery. Previous cardiac surgery.	Symptoms (exertional dyspnoea, syncope, presyncope or angina). LVEF <50%. Clinically significant aortic regurgitation or mitral valve disease. Previous cardiac surgery.
Aetiology of aortic stenosis	Degenerative valvular disease: 133 patients (84.7%). Bicuspid aortic valve: 22 patients (14.0%). Rheumatic valvular disease: 2 patients (1.3%).	Degenerative valvular disease: 48 (33%). Bicuspid aortic valve: 88 patients (61%). Rheumatic valvular disease: 9 patients (6%).
Primary endpoints	All-cause mortality or major adverse cardiovascular events comprised all-cause death, acute myocardial infarction, stroke and unplanned heart failure hospitalisation needing intravenous treatment with diuretics or inotropes.	Operative mortality (death during or within 30 days after surgery) or death from cardiovascular causes during the entire follow-up period.

Data are presented as available by the relevant published studies.

AS, aortic stenosis; AVA, aortic valve area; AVATAR, Aortic Valve Replacement versus Conservative Treatment in Asymptomatic Severe Aortic Stenosis; LVEF, left ventricular ejection fraction; MG, mean gradient; RECOVERY, Randomized Comparison of Early Surgery versus Conventional Treatment in Very Severe Aortic Stenosis; Vmax, maximal velocity across the aortic valve.

**Table 2:** Baseline demographic and echocardiographic characteristics of the patient population of the two randomised controlled trials

	AVATAR		RECOVERY	
	Early surgery	Conservative care	Early surgery	Conservative care
Number of participants	78	79	73	72
Age (years)	68 (63–73)*	69 (64–74.5)*	65±7.8†	63.4±10.7†
Sex (male, %)	46 (59)	44 (55.7)	37 (51)	34 (47)
Median follow-up (months)	28	35	74.4	73.2
Demographic parameters				
BMI (kg/m^2^)	27.2	27.4	24.7	24
BSA (m^2^)	1.9 (1.8–2.1)*	1.9 (1.8–2.0)*	1.69±0.17†	1.64±0.17†
Diabetes mellitus, n (%)	14 (17.9)	23 (29.1)	13 (18)	7 (10)
Hypertension, n (%)	69 (88.4)	70 (88.6)	40 (55)	39 (54)
Smoking, n (%)	16 (20.5)	14 (17.7)	19 (26)	21 (29)
Dyslipidaemia, n (%)	31 (39.7)	28 (35.4)	41 (56)	42 (58)
Echocardiographic parameters				
AVA, cm^2^	0.73*	0.74*	0.63†	0.64†
AVAi, cm^2^/m^2^	0.37*	0.37*	0.38†	0.39†
Vmax, m/s	4.5*	4.5*	5.14†	5.04†
LVEF (%)	70*	69*	64.8†	64.8†
LV mass index, g/m²	152*	160*	135.6†	133.7†

Data are presented as available by the relevant published studies.

*Median (IQR).

†Mean±SD.

AVA, aortic valve area; AVAi, indexed aortic valve area; AVATAR, Aortic Valve Replacement versus Conservative Treatment in Asymptomatic Severe Aortic Stenosis; BMI, body mass index; BSA, body surface area; LV, left ventricular; LVEF, left ventricular ejection fraction; RECOVERY, Randomized Comparison of Early Surgery versus Conventional Treatment in Very Severe Aortic Stenosis; Vmax, maximal velocity across the aortic valve.

Despite the different endpoints of the trials, both demonstrated a reduced number of deaths from any cause in the group of patients treated with early surgery. More specifically, in the AVATAR trial there were 9 deaths from any cause in the early intervention group and 16 deaths in the conservative management group. Similarly in the RECOVERY trial there were 5 deaths in the early intervention group and 15 deaths in the conservative management group. There was a similar pattern in the number of hospitalisations for heart failure, with reduced number of hospitalisations in the early intervention groups in both trials compared with the conservative management groups. The raw number of events provided by each trial for all-cause death, hospitalisation for heart failure, myocardial infarction and stroke is depicted in online supplemental table 2.

**Figure 1:**
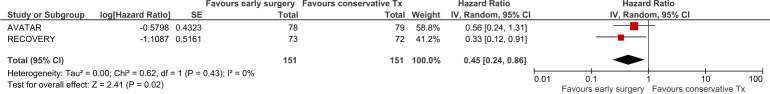
Meta-analysis of AVATAR and RECOVERY trials focusing on all-cause mortality: the effect of early intervention on all-cause mortality. AVATAR, Aortic Valve Replacement versus Conservative Treatment in Asymptomatic Severe Aortic Stenosis. RECOVERY, Randomized Comparison of Early Surgery versus Conventional Treatment in Very Severe Aortic Stenosis; IV, interval variable; Tx, treatment.

**Figure 2:**
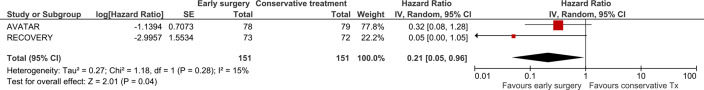
Meta-analysis of AVATAR and RECOVERY trials focusing on hospitalisation for heart failure. Early intervention resulted in significant risk reduction of hospitalisation for heart failure. AVATAR, Aortic Valve Replacement versus Conservative Treatment in Asymptomatic Severe Aortic Stenosis. RECOVERY, Randomized Comparison of Early Surgery versus Conventional Treatment in Very Severe Aortic Stenosis. IV, interval variable; Tx, treatment.

The pooled meta-analysis showed a significant reduction in all-cause mortality in patients treated with the early intervention compared with those managed conservatively with the watchful waiting approach (HR=0.45, 95% CI 0.24 to 0.86; I^2^ 0%) (figure 1). Furthermore, the early intervention resulted in a significantly reduced risk of hospitalisation for heart failure (HR=0.21, 95% CI 0.05 to 0.96; I^2^ 15%) (figure 2). There was significant heterogeneity in the meta-analysis of cardiovascular mortality among the two studies, and while there was a trend towards improved survival this was mainly driven by the RECOVERY trial and did not reach statistical significance (HR=0.36, 95% CI 0.03 to 3.78; I^2^ 78%) (online supplemental figure 2).

### Meta-analysis of observational studies

Additionally, eight observational studies, including 3496 patients, were analysed separately to assess how early intervention affects the risk of all-cause mortality in patients with asymptomatic severe aortic stenosis.^7 11–17^ Table 3 summarises the main characteristics of each study, while the risk of bias assessment is demonstrated in online supplemental table 3.

**Table 3:** Characteristics of observational studies comparing early intervention versus conservative management

Study	Study design	Study population	Age (years)	Follow-up period	Outcomes	Adjustment methods
Pai *et al*^11^	Retrospective cohort study	338	71±15	7.6 months	All-cause mortality.	Cox proportional hazards model (variables adjusted for age, MR grade 3 or 4, CKD and aspirin use).
Kang *et al*^12^	Prospective cohort study	104	63±12	49.3 months	Operative mortality and cardiovascular death during follow-up.	Propensity score-matched analysis.
Le Tourneau *et al*^13^	Retrospective cohort study	694	71±11	Average more than 60 months	To assess the value of STS score in predicting short-term and long-term outcome.	Cox proportional hazards model.
Taniguchi *et al* (CURRENT AS registry)^7^	Retrospective cohort study (propensity score-matched analysis)	582	71.6±8.7 (AVR group) 73.1±9.3 (watchful waiting group)	45 months (median)	All-cause mortality and hospitalisation for heart failure.	Propensity score-matched analysis.
Masri *et al*^14^	Retrospective cohort study	533	66±13	82.8 months	All-cause mortality.	Cox proportional hazards model (variables adjusted for STS score, age-sex-predicted METs and heart rate recovery).
Bohbot *et al*^15^	Retrospective cohort study	281	73±10	42 months	All-cause mortality and cardiovascular death during follow-up.	Cox proportional hazards model (variables adjusted for age, sex, body surface area, hypertension, coronary artery disease, atrial fibrillation, Charlson Comorbidity Index, AVA, peak aortic jet velocity, EF and LV mass).
Campo *et al*^16^	Retrospective cohort study	265	68.1±11.7 (AVR group) 73±12.6 (watchful waiting group)	60 months	All-cause mortality.	Multivariable Cox regression model (variables adjusted for age, renal failure and ejection fraction).
Kim *et al*^17^	Retrospective cohort study	468	64.2±13	60.9 months	All-cause mortality, cardiovascular death and MACE.	Time-dependent Cox regression analysis (variables adjusted for age, BMI, anaemia, severe CKD, previous stroke, coronary artery disease, previous malignancy, left atrium diameter, LVMi and tricuspid regurgitation pressure gradient).

AVR, aortic valve replacement; BMI, body mass index; CKD, chronic kidney disease; CURRENT AS, Contemporary outcomes after Surgery and medical treatment in patients with severe Aortic Stenosis; EF, ejection fraction; LV, left ventricular; LVMi, left ventricular mass indexed; MACE, major adverse cardiac events; METs, metabolic equivalents; MR, mitral regurgitation; STS, Society of Thoracic Surgeons.

**Figure 3:**
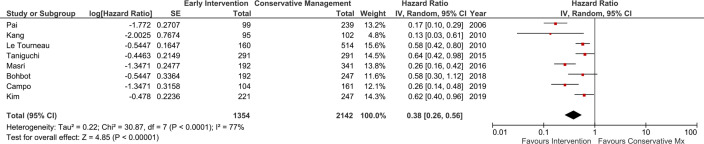
Meta-analysis of observational studies: impact of early aortic valve intervention versus conservative management on all-cause mortality. IV, interval variable; Mx, management.

Of the total population, 1354 patients had an early aortic valve replacement and 2142 were managed conservatively with the watchful waiting approach. The meta-analysis from these studies showed that early intervention was associated with a 62% reduction in all-cause mortality (HR=0.38, 95% CI 0.26 to 0.56), although heterogeneity was high (I^2^ 77%) (figure 3). Online supplemental figure 3 shows the funnel plot for risk of all-cause mortality from these eight studies.

## Discussion

Our report represents the first meta-analysis of the two published randomised controlled trials assessing the impact of early intervention on the outcomes of patients with asymptomatic severe aortic stenosis. It demonstrates that all-cause mortality and hospitalisation for heart failure are significantly lower in patients randomised to early surgery compared with those treated with an initial conservative approach. Regarding cardiovascular mortality, while there was a trend towards improved survival in the early intervention group, this was mainly driven by the RECOVERY trial and was not found to be significantly different between the early and conservative groups. Additionally, a meta-analysis of eight observational studies showed a notable reduction in all-cause mortality in the early intervention group. This is in agreement with previous meta-analyses that were conducted even before the AVATAR trial was published, which showed a favourable outcome in those treated with early intervention versus conservative management.^18^

The timing of intervention in patients with severe aortic stenosis remains contentious. The lack of large randomised controlled studies has been a ‘deafening silence’ in cardiology, with current practice often informed by observational studies, which despite their limitations timidly show a favour towards early intervention. Nonetheless, this needs to be balanced by the potentially longer need for a valve replacement, which in itself can come with risks of bleeding, endocarditis and failure. Several unanswered questions have long been discussed among clinicians. Current guidelines suggest intervention in patients with severe aortic stenosis when symptoms occur or in those without symptoms and left ventricular ejection fraction (LVEF) <50% or abnormal exercise test or Vmax >5 m/s or brain natriuretic peptide (BNP) more than three times the upper normal value or features of rapid disease progression.^3 19 20^ However, it can be challenging to unmask these on many occasions, especially in patients with sedentary life or in the frail elderly population. Additionally, the presence of left ventricular impairment represents a high-risk feature that mandates intervention in these patients. Still, some argue that it may already be too late when the LVEF drops.^21 22^ So far, there has been an ongoing debate with studies demonstrating a low risk of death in patients with asymptomatic aortic stenosis unless there is very severe aortic stenosis or signs of rapid progression or myocardial impairment.^23 24^ Nevertheless, a retrospective observational study by Campo *et al*^16^ demonstrated that almost half of the asymptomatic patients with severe aortic stenosis would have either died or undergone surgery within 2 years of the initial recommendation for the watchful waiting strategy.^15^ This is in keeping with another retrospective registry by Pellikka *et al*,^25^ stating that 75% of these patients will have died or have been operated on 5 years after the diagnosis. However, evidence suggests that, although this risk is low, it is still higher than the general population and this clinical entity may not be as benign as previously thought.^25 26^

The two recently published studies, AVATAR and RECOVERY trials, have been enthusiastically awaited to answer some of these questions. Arguably, the two trials, while they do have differences as mentioned in table 2, both demonstrate a consistent benefit of the early intervention approach. However, there are essential points that need to be considered. The two trials have had some important differences in their study characteristics, with the RECOVERY trial including patients with more severe aortic stenosis. This could be one of the reasons why the RECOVERY trial demonstrated a more pronounced reduction in mortality and morbidity in favour of the early intervention strategy, as it is known that these patients are at higher risk of death and adverse prognosis.^27 28^ One of the most notable things, however, is that rapid progression of aortic stenosis in the RECOVERY trial was defined as a change of 0.5 m/s per year, which is in contrast to what the current^4^ and the previous guidelines,^29^ on which the trial was based, defined as rapid progression of the disease. This, of course, raises the question as to whether there were participants in the conservative arm that fulfilled the criteria for intervention. Moreover, the patient population in the RECOVERY trial consisted mainly of patients with aortic stenosis secondary to the bicuspid aortic valve. Subsequently, participants of this trial were younger with low operative risk compared with the AVATAR trial.^10^ On the other hand, the majority of the patients included in the AVATAR trial had aortic stenosis secondary to degenerative disease. However, the operative risk was low as it was in the RECOVERY trial.^8^ Furthermore, while in the RECOVERY trial exercise test was used in selected cases only, all participants of the AVATAR trial underwent an exercise test to unmask symptoms or adverse signs before their inclusion. However, it is unclear if and how the exercise test was used in the AVATAR trial during the follow-up of these patients.

In addition to the above, whereas this meta-analysis showed a significant reduction in all-cause mortality in the early intervention group, this was not the case for cardiovascular death, where high heterogeneity was noted and no significant difference was demonstrated between the two management strategies. While the issues discussed above may have contributed to this discrepancy, perhaps one of the most important factors that should be considered is the follow-up period. The follow-up periods of the two studies are substantially different, with the median follow-up of the RECOVERY trial reaching 6.1 years, more than twice the AVATAR trial follow-up. On close examination, it is noted that the incidence of cardiovascular death between the two treatment groups in the RECOVERY trial becomes substantially different after approximately the first 2.5 years of the follow-up period. In the conservative group the cumulative incidence of cardiovascular death rises significantly, reaching a peak at 8 years after randomisation, while the intervention group demonstrates only a small rise.^10^ Taking into consideration that the median follow-up of the conservative group in the AVATAR trial was just over 2.5 years (35 months), one could argue that a difference in the incidence of cardiovascular death between the two trials is expected. Previous data from a retrospective registry suggest that mortality between the two groups of patients (those treated with early surgery and those conservatively managed) begins to significantly differ after 1 year of follow-up.^13^

It is important to note that both studies were performed at different time points in the past. Therefore, the inclusion criteria and characteristics of the study population may not reflect the most recent guidelines and recommendations. For instance, according to the latest European Society of Cardiology (ESC) guidelines, intervention should be considered in asymptomatic patients with low operative risk, LVEF >55% and Vmax ≥5 m/s or in asymptomatic individuals with LVEF <55% without another cause (class IIa recommendation).^3^ Given the inclusion and exclusion criteria of the AVATAR and RECOVERY trials, some of the participants included in both studies unavoidably fall in these categories.

The meta-analysis from the observational studies yielded a categorically favourable outcome of the early intervention group compared with the conservative management. However, given their non-randomised design, the risk of bias is high. Therefore, conclusions should be drawn cautiously. Additionally, there was considerable heterogeneity in the meta-analysis from these studies that could be attributed to the different study designs and analysis methods, inclusion criteria and baseline patient characteristics, and different follow-up periods.

Finally, when putting the findings of these meta-analyses into clinical context, the baseline characteristics of the patient population included, and more specifically the age, should be taken into consideration. The participants of both trials and of some of the observational studies were relatively young, with a mean age of less than 70 years old. This may not fully reflect the cohort of patients usually seen in secondary or tertiary care, which may consist of older patients with more comorbidities and therefore higher operative risk.^30^

Despite differences and limitations of the studies, all the data from this meta-analysis would support consideration of an earlier approach and highlight, however given the differences and limitations in the two studies, it would be prudent to allow the ongoing research in the field to complete. Nonetheless, this meta-analysis gives us an early insight as to what outcome we could expect. It is noteworthy that in a large registry that included more than 25 000 patients with aortic stenosis, the individuals with moderate aortic stenosis exhibited a surprisingly high risk of all-cause and cardiovascular deaths, which was similar to those with severe aortic stenosis.^31^ Whether this finding means that some patients with parameters in the moderate spectrum of the disease are at high risk of death from the valvular disease alone or that, inevitably, comorbidities in this cohort play a much more significant role than previously thought remains unclear. Nevertheless, in the era of personalised medicine, the identification of the high-risk population and the decision regarding intervention should be made after careful individual risk assessment and evaluation not only of symptoms but also of indirect signs of myocardial decompensation and damage that could have a negative impact on the prognosis and outcome. Such parameters could include the presence of mid-wall late gadolinium enhancement of cardiovascular magnetic resonance, utilisation of left ventricular stroke volume in preference to LVEF or various biomarkers (eg, N-terminal pro b-type natriuretic peptide/BNP, high-sensitivity troponin), which are associated with a worse outcome.^32–35^ These clues could help refine the optimal timing of intervention for each patient.^35–37^ The results of currently ongoing randomised trials investigating these parameters are greatly anticipated to help clarify the role of these parameters in the risk assessment of patients with severe aortic stenosis and the impact of early intervention on the outcome and prognosis. The main characteristics of these trials are summarised in table 4.

**Table 4:** Main study characteristics of the ongoing randomised controlled trials

	EASY-AS^38^	EARLY TAVR^39^	DANAVR^40^	EVoLVeD^41^
Identifier	NCT04204915	NCT03042104	NCT03972644	NCT03094143
Estimated enrolment	2844 participants	900 participants	1700 participants	1000 participants
Estimated completion date	October 2029	March 2024	September 2029	October 2024
Intervention	AVR	TAVR	SAVR or TAVR	SAVR or TAVR (participants will be randomised based on the presence or absence of fibrosis on CMR)
Primary outcomes	Cardiovascular death and hospitalisation for heart failure	All-cause death, all stroke and unplanned cardiovascular hospitalisation	All-cause mortality	All-cause mortality or unplanned aortic stenosis-related hospitalisation
Key inclusion criteria	Asymptomatic severe AS. Age >18 years. LVEF ≥50%.	Asymptomatic severe AS. Age ≥65 years. LVEF ≥50%. STS risk score <10.	Asymptomatic severe AS. Age ≥18 and ≤85 years. LVEF ≥50%.	Asymptomatic severe AS. Age >18 years. LVEF ≥50% on CMR.

AS, aortic stenosis; AVR, aortic valve replacement; CMR, cardiac magnetic resonance; DANAVR, Danish National Randomized Study on Early Aortic Valve Replacement in Patients with Asymptomatic Severe Aortic Stenosis; EARLY-TAVR, Evaluation of TAVR Compared to Surveillance for Patients with Asymptomatic Severe Aortic Stenosis; EASY-AS, Early Valve Replacement in Severe Asymptomatic Aortic Stenosis Study; EVoLVeD, Early Valve Replacement Guided by Biomarkers of LV Decompensation in Asymptomatic Patients with Severe AS; LVEF, left ventricular ejection fraction; SAVR, surgical aortic valve replacement; STS, Society of Thoracic Surgeons; TAVR, transcatheter aortic valve replacement.

## Conclusion

Asymptomatic severe aortic stenosis represents a unique entity that poses clinical dilemmas to physicians worldwide very frequently in clinical practice. This meta-analysis shows that the data from the two recent large randomised controlled trials and previous observational studies demonstrate a favourable outcome in the group of patients treated with early intervention rather than conservative management. Although this may herald the beginning of a change in the management of these patients, further randomised controlled studies are needed to draw firm conclusions and identify the optimal timing of intervention.

## Data Availability

All data relevant to the study are included in the article or uploaded as supplementary information.

## References

[1] Coffey S, Cairns BJ, Iung B. The modern epidemiology of heart valve disease. Heart 2016;102:75–85. 10.1136/heartjnl-2014-307020 26541169

[2] Coffey S, Cox B, Williams MJA. Lack of progress in valvular heart disease in the pre-transcatheter aortic valve replacement era: increasing deaths and minimal change in mortality rate over the past three decades. Am Heart J 2014;167:562–7. 10.1016/j.ahj.2013.12.030 24655706

[3] Vahanian A, Beyersdorf F, Praz F, et al. 2021 ESC/EACTS guidelines for the management of valvular heart disease. Eur Heart J 2022;43:561–632. 10.1093/eurheartj/ehab395 34453165

[4] Otto CM, Nishimura RA, Bonow RO, et al. 2020 ACC/AHA guideline for the management of patients with valvular heart disease: a report of the American College of Cardiology/American Heart Association Joint Committee on Clinical Practice Guidelines. Circulation 2021;143:e72–227. 10.1161/CIR.0000000000000923 33332150

[5] Bing R, Everett RJ, Tuck C, et al. Rationale and design of the randomized, controlled early valve replacement guided by biomarkers of left ventricular decompensation in asymptomatic patients with severe aortic stenosis (EVOLVED) trial. Am Heart J 2019;212:91–100. 10.1016/j.ahj.2019.02.018 30978556

[6] Heuvelman HJ, van Geldorp MWA, Kappetein AP, et al. Clinical course of patients diagnosed with severe aortic stenosis in the Rotterdam area: insights from the AVARIJN study. Neth Heart J 2012;20:487–93. 10.1007/s12471-012-0309-3 22864980 PMC3515726

[7] Taniguchi T, Morimoto T, Shiomi H, et al. Initial surgical versus conservative strategies in patients with asymptomatic severe aortic stenosis. J Am Coll Cardiol 2015;66:2827–38. 10.1016/j.jacc.2015.10.001 26477634

[8] Banovic M, Putnik S, Penicka M, et al. Aortic valve replacement versus conservative treatment in asymptomatic severe aortic stenosis: the AVATAR trial. Circulation 2022;145:648–58. 10.1161/CIRCULATIONAHA.121.057639 34779220

[9] Page MJ, McKenzie JE, Bossuyt PM, et al. The PRISMA 2020 statement: an updated guideline for reporting systematic reviews. BMJ 2021;372:n71. 10.1136/bmj.n71 33782057 PMC8005924

[10] Kang D-H, Park S-J, Lee S-A, et al. Early surgery or conservative care for asymptomatic aortic stenosis. N Engl J Med 2020;382:111–9. 10.1056/NEJMoa1912846 31733181

[11] Pai RG, Kapoor N, Bansal RC, et al. Malignant natural history of asymptomatic severe aortic stenosis: benefit of aortic valve replacement. Ann Thorac Surg 2006;82:2116–22. 10.1016/j.athoracsur.2006.07.043 17126122

[12] Kang D-H, Park S-J, Rim JH, et al. Early surgery versus conventional treatment in asymptomatic very severe aortic stenosis. Circulation 2010;121:1502–9. 10.1161/CIRCULATIONAHA.109.909903 20308614

[13] Le Tourneau T, Pellikka PA, Brown ML, et al. Clinical outcome of asymptomatic severe aortic stenosis with medical and surgical management: importance of STS score at diagnosis. Ann Thorac Surg 2010;90:1876–83. 10.1016/j.athoracsur.2010.07.070 21095330

[14] Masri A, Goodman AL, Barr T, et al. Predictors of long-term outcomes in asymptomatic patients with severe aortic stenosis and preserved left ventricular systolic function undergoing exercise echocardiography. Circ Cardiovasc Imaging 2016;9:1–9. 10.1161/CIRCIMAGING.116.004689 27406843

[15] Bohbot Y, Pasquet A, Rusinaru D, et al. Asymptomatic Severe Aortic Stenosis With Preserved Ejection Fraction: Early Surgery Versus Conservative Management. J Am Coll Cardiol 2018;72:2938–9. 10.1016/j.jacc.2018.09.049 30522658

[16] Campo J, Tsoris A, Kruse J, et al. Prognosis of severe asymptomatic aortic stenosis with and without surgery. Ann Thorac Surg 2019;108:74–9. 10.1016/j.athoracsur.2019.01.031 30905426

[17] Kim HJ, Kim JB, Kim HR, et al. Impact of valve replacement on long-term survival in asymptomatic patients with severe aortic stenosis. Am J Cardiol 2019;123:1321–8. 10.1016/j.amjcard.2019.01.035 30745019

[18] Yuan T, Lu Y, Bian C, et al. Early aortic valve replacement vs. conservative management in asymptomatic severe aortic stenosis patients with preserved ejection fraction: a meta-analysis. Front Cardiovasc Med 2020;7:1–9. 10.3389/fcvm.2020.621149 33614743 PMC7887283

[19] , Otto CM, Nishimura RA, et al, Writing Committee Members. 2020 ACC/AHA guideline for the management of patients with valvular heart disease: Executive summary: a report of the American College of Cardiology/American heart association joint Committee on clinical practice guidelines. J Am Coll Cardiol 2021;77:450–500. 10.1016/j.jacc.2020.11.035 33342587

[20] N. Guideline scope. Heart valve disease presenting in adults: investigation and management. Nice, 2019: 1–12. 35143140

[21] Perry AS, Li S. Optimal threshold of left ventricular ejection fraction for aortic valve replacement in asymptomatic severe aortic stenosis: a systematic review and meta-analysis. J Am Heart Assoc 2021;10:e020252. 10.1161/JAHA.120.020252 33787311 PMC8174345

[22] Taniguchi T, Morimoto T, Takeji Y, et al. Contemporary issues in severe aortic stenosis: review of current and future strategies from the contemporary outcomes after surgery and medical treatment in patients with severe aortic stenosis registry. Heart 2020;106:802–9. 10.1136/heartjnl-2019-315672 32114519

[23] Lancellotti P, Magne J, Dulgheru R, et al. Outcomes of patients with asymptomatic aortic stenosis followed up in heart valve clinics. JAMA Cardiol 2018;3:1060–8. 10.1001/jamacardio.2018.3152 30285058 PMC6583052

[24] Rosenhek R, Binder T, Porenta G, et al. Predictors of outcome in severe, asymptomatic aortic stenosis. N Engl J Med 2000;343:611–7. 10.1056/NEJM200008313430903 10965007

[25] Pellikka PA, Sarano ME, Nishimura RA, et al. Outcome of 622 adults with asymptomatic, hemodynamically significant aortic stenosis during prolonged follow-up. Circulation 2005;111:3290–5. 10.1161/CIRCULATIONAHA.104.495903 15956131

[26] Kvaslerud AB, Santic K, Hussain AI, et al. Outcomes in asymptomatic, severe aortic stenosis. PLoS One 2021;16:e0249610. 10.1371/journal.pone.0249610 33826652 PMC8026050

[27] Saito T, Muro T, Takeda H, et al. Prognostic value of aortic valve area index in asymptomatic patients with severe aortic stenosis. Am J Cardiol 2012;110:93–7. 10.1016/j.amjcard.2012.02.056 22497679

[28] Nishimura S, Izumi C, Nishiga M, et al. Predictors of rapid progression and clinical outcome of asymptomatic severe aortic stenosis. Circ J 2016;80:1863–9. 10.1253/circj.CJ-16-0333 27334030

[29] American College of Cardiology/American Heart Association Task Force on Practice Guidelines, Society of Cardiovascular Anesthesiologists, Society for Cardiovascular Angiography and Interventions, et al. ACC/AHA 2006 guidelines for the management of patients with valvular heart disease: a report of the American College of Cardiology/American heart association Task force on practice guidelines (writing Committee to revise the 1998 guidelines for the management of patients with valvular heart disease): developed in collaboration with the Society of cardiovascular Anesthesiologists: endorsed by the Society for cardiovascular angiography and interventions and the Society of thoracic surgeons. Circulation 2006;114:e84–231. 10.1161/CIRCULATIONAHA.106.176857 16880336

[30] Taylor CJ, Ordóñez-Mena JM, Jones NR, et al. Survival of people with valvular heart disease in a large, English community-based cohort study. Heart 2021;107:1336–43. 10.1136/heartjnl-2020-318823 34031157 PMC8327406

[31] Strange G, Stewart S, Celermajer D, et al. Poor long-term survival in patients with moderate aortic stenosis. J Am Coll Cardiol 2019;74:1851–63. 10.1016/j.jacc.2019.08.004 31491546

[32] Vassiliou VS, Perperoglou A, Raphael CE, et al. Midwall fibrosis and 5-year outcome in moderate and severe aortic stenosis. J Am Coll Cardiol 2017;69:1755–6. 10.1016/j.jacc.2017.01.034 28359524

[33] Kuk M, Newsome S, Alpendurada F, et al. A model based on clinical parameters to identify myocardial late gadolinium enhancement by magnetic resonance in patients with aortic stenosis: an observational study. JRSM Cardiovasc Dis 2020;9:204800402092240. 10.1177/2048004020922400 PMC7218314 32426125

[34] Vassiliou VS, Pavlou M, Malley T, et al. A novel cardiovascular magnetic resonance risk score for predicting mortality following surgical aortic valve replacement. Sci Rep 2021;11:20183. 10.1038/s41598-021-99788-7 34642428 PMC8511276

[35] White M, Baral R, Ryding A, et al. Biomarkers associated with mortality in aortic stenosis: a systematic review and meta-analysis. Med Sci 2021;9:29. 10.3390/medsci9020029 PMC8163007 34067808

[36] Kwak S, Everett RJ, Treibel TA, et al. Markers of myocardial damage predict mortality in patients with aortic stenosis. J Am Coll Cardiol 2021;78:545–58. 10.1016/j.jacc.2021.05.047 34353531

[37] Bing R, Dweck MR. Management of asymptomatic severe aortic stenosis: check or all in? Heart 2021;107:842–50. 10.1136/heartjnl-2020-317160 33148547

[38] ClinicalTrials.gov. The Early Valve Replacement in Severe ASYmptomatic Aortic Stenosis Study - Full Text View -. Available: https://clinicaltrials.gov/ct2/show/study/NCT04204915?term=ASYMPTOMATIC&cond=AORTIC+STENOSIS&draw=3&rank=2 [Accessed 1 Dec 2021].

[39] ClinicalTrials.gov. EARLY TAVR: Evaluation of TAVR Compared to Surveillance for Patients With Asymptomatic Severe Aortic Stenosis - Full Text View-. Available: https://clinicaltrials.gov/ct2/show/NCT03042104?term=ASYMPTOMATIC&cond=AORTIC+STENOSIS&draw=3&rank=5 [Accessed 1 Dec 2021].

[40] ClinicalTrials.gov. Danish National Randomized Study on Early Aortic Valve Replacement in Patients With Asymptomatic Severe Aortic Stenosis - Full Text View -. Available: https://clinicaltrials.gov/ct2/show/NCT03972644?term=ASYMPTOMATIC&cond=AORTIC+STENOSIS&draw=3&rank=8 [Accessed 1 Dec 2021].

[41] ClinicalTrials.gov. Early Valve Replacement Guided by Biomarkers of LV Decompensation in Asymptomatic Patients With Severe AS - Full Text View -. Available: https://clinicaltrials.gov/ct2/show/NCT03094143?term=ASYMPTOMATIC&cond=AORTIC+STENOSIS&draw=3&rank=12 [Accessed 1 Dec 2021].

