# Supplementary material for: Management of asymptomatic severe aortic stenosis: a systematic review and meta-analysis
Source: Open Heart. 2022 May 12;9(1):e001982. doi: 10.1136/openhrt-2022-001982 (PMC9109115; doi:10.1136/openhrt-2022-001982)
Supplement: Supplementary data [file openhrt-2022-001982supp001.pdf]

BMJ Publishing Group Limited (BMJ) disclaims all liability and responsibility arising from any reliance placed on this supplemental material which has been supplied by the author(s)

Management of asymptomatic severe aortic stenosis: A systematic review and meta-analysis

Vasiliki Tsampasian[1,2], Ciaran Grafton-Clarke[1,2], Abraham Edgar Gracia Ramos[3], George Asimakopoulos[4], Pankaj Garg[1,2], Sanjay K Prasad[4], Liam Ring[5], Gerry McCann[6], James H F Rudd[7], Marc R Dweck[8], Vassilios S Vassiliou[1,2]

Supplementary Material

Supplementary Figure 1. PRISMA flow diagram of the study selection process.

Supplementary Figure 2. Meta-analysis of AVATAR and RECOVERY trials focusing on cardiovascular death. No statistically significant differences were seen between the two treatment groups.

Supplementary Figure 3. Funnel plot for risk of all-cause mortality as derived from the eight observational studies demonstrating no evidence of significant publication bias

Supplementary Table 1. Cochrane Collaboration's tool for assessing risk of bias in randomised controlled trials

Supplementary Table 2. Number of events in each randomised controlled trial

Supplementary Table 3. Newcastle-Ottawa Scale for non-randomised clinical trials

Supplementary Figure 1. PRISMA flow diagram of the study selection process.

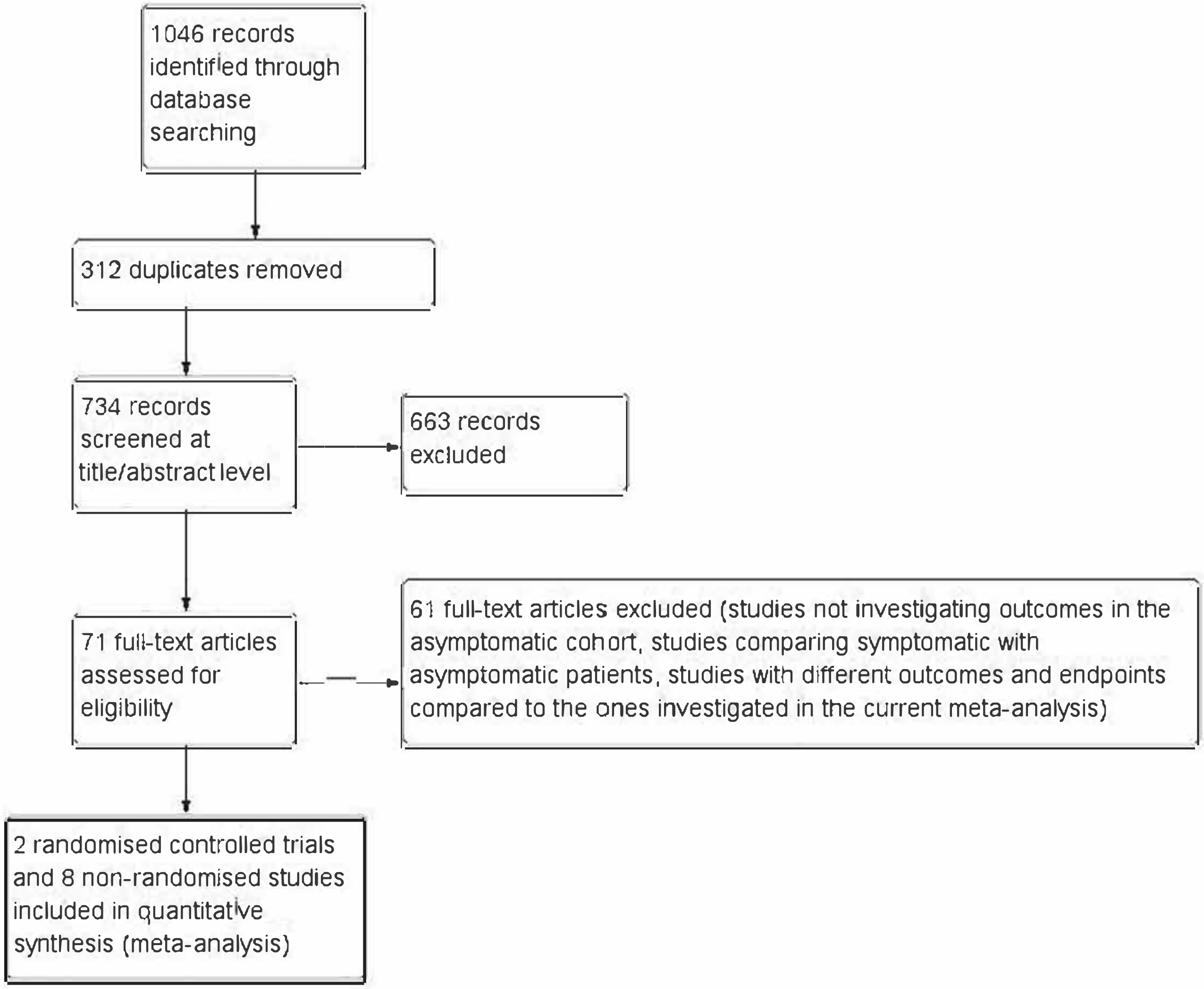

Supplementary Figure 2. Meta-analysis of AVATAR and RECOVERY trials focusing on cardiovascular death. No statistically significant differences were seen between the two treatment groups.

Study or Subgroup	log[Hazard Ratio]	SE	Early Surgery Total	Conservative Treatment Total	Weight	Hazard Ratio IV, Random, 95% CI
AVATAR	0.0198	0.4776	78	79	57.0%	1.02 [0.40, 2.60]
RECOVERY	-2.4079	1.0242	73	72	43.0%	0.09 [0.01, 0.67]
**Total (95% CI)**			**151**	**151**	**100.0%**	**0.36 [0.03, 3.78]**

Heterogeneity: Tau² = 2.31; Chi² = 4.61, df = 1 (P = 0.03); I² = 78%
Test for overall effect: Z = 0.85 (P = 0.39)

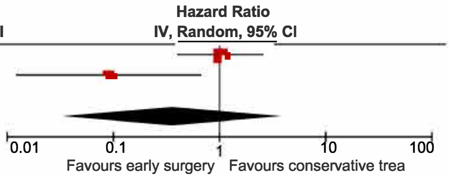

Supplementary Figure 3. Funnel plot for risk of all-cause mortality as derived from the eight observational studies demonstrating no evidence of significant publication bias

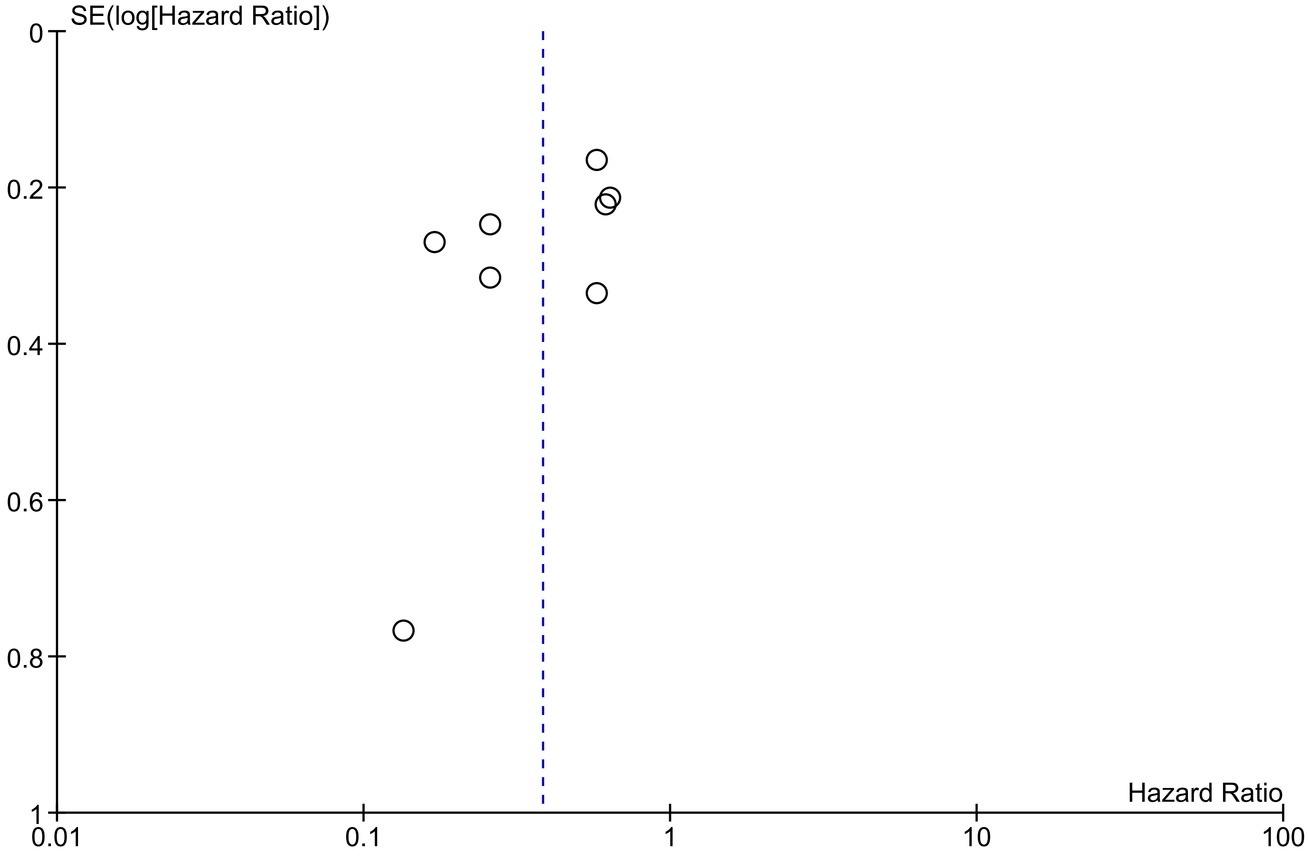

Supplementary Table 1. Cochrane Collaboration's tool for assessing risk of bias in randomised controlled trials

**Trial**	**Sequence generation**	**Allocation concealment**	**Performance bias**	**Detection bias**	**Attrition bias**	**Reporting bias**
**AVATAR**	Low	Low	Low	Low	Low	Low
**RECOVERY**	Low	Low	Low	Low	Low	Low

Supplementary Table 2. Number of events in each randomised controlled trial

	AVATAR		RECOVERY	
	Early surgery	Conservative care	Early surgery	Conservative care
All-cause death - No (%)	9 (11.5%)	16 (20.2%)	5 (6.8%)	15 (20.8%)
Heart failure hospitalisation - No (%)	1 (1.2%)	7 (8.8%)	0 (0%)	8 (11.1%)
Myocardial infarction - No (%)	1 (1.2%)	2 (2.5%)	0 (0%)	1 (1.4%)
Stroke - No (%)	2 (2.5%)	1 (1.2%)	1 (1.3%)	3 (4.2%)

Supplementary Table 3. Newcastle-Ottawa Scale for non-randomised clinical trials

Study	Selection	Comparability	Outcome	Total	Quality of study
**Pai**	****	*	**	7	High
**Kang**	****	*	***	8	High
**Le Tourneau**	****	**	**	7	High
**Taniguchi (CURRENT AS)**	****	**	***	9	High
**Masri**	***	*	***	7	High
**Bohbot**	****	*	***	8	High
**Campo**	****	*	**	7	High
**Kim**	****	*	***	8	High
